# Pancreas Optical Clearing and 3-D Microscopy in Health and Diabetes

**DOI:** 10.3389/fendo.2021.644826

**Published:** 2021-04-26

**Authors:** Martha Campbell-Thompson, Shiue-Cheng Tang

**Affiliations:** ^1^ Department of Pathology, Immunology and Laboratory Medicine, College of Medicine, University of Florida, Gainesville, FL, United States; ^2^ Department of Medical Science and Institute of Biotechnology, National Tsing Hua University, Hsinchu, Taiwan

**Keywords:** islet, autonomic (vegetative) nervous system, lightsheet microscopy, CLARITY, adipocyte, Schwann cell, confocal 3-D microscopy, organoid

## Abstract

Although first described over a hundred years ago, tissue optical clearing is undergoing renewed interest due to numerous advances in optical clearing methods, microscopy systems, and three-dimensional (3-D) image analysis programs. These advances are advantageous for intact mouse tissues or pieces of human tissues because samples sized several millimeters can be studied. Optical clearing methods are particularly useful for studies of the neuroanatomy of the central and peripheral nervous systems and tissue vasculature or lymphatic system. Using examples from solvent- and aqueous-based optical clearing methods, the mouse and human pancreatic structures and networks will be reviewed in 3-D for neuro-insular complexes, parasympathetic ganglia, and adipocyte infiltration as well as lymphatics in diabetes. Optical clearing with multiplex immunofluorescence microscopy provides new opportunities to examine the role of the nervous and circulatory systems in pancreatic and islet functions by defining their neurovascular anatomy in health and diabetes.

## Introduction

### Goals

Heterogeneity of the human pancreas is well accepted in terms of islet endocrine cell proportions and mass in healthy people and for lobularity in islet beta-cell losses and frequency of infiltrated islets in patients in type 1 diabetes (T1D) (1–7). Patients with type 2 diabetes (T2D) show similar heterogeneity in islet amyloidosis, fatty infiltration, fibrosis and inflammatory infiltrates (4, 8–10). Morphology-based studies of the human pancreas have been key to understanding regional heterogeneity yet examinations of the pancreas in its natural three-dimensional (3-D) configuration have been limited to laborious serial sectioning with subsequent reconstruction. Islets occupy only ~2% of the entire pancreas volume and sampling of multiple blocks is recommended to maximize islet analyzes by 2-D microscopy (11). Recent applications of optical clearing methods to the human and mouse pancreas provide new details for structure-function relationships in health and subsequent abnormalities in diabetes (12–15). This review provides an overview of recent optical clearing methods used in human and mouse pancreas studies and examples of pancreas optical clearing to define several components of the pancreas endocrine and exocrine compartments.

## Techniques

Although basic optical clearing to render tissues transparent was first described by the German anatomist Walter Spalteholz over 100 years ago, the recent decade has seen a rapid growth in advanced clearing methods applicable for whole body or organ imaging to single cell resolution (16, 17). Optical clearing is readily accomplished in a standard laboratory and new procedures can be found nearly weekly in the literature for different organs and species (**Table 1**). Optical clearing methods are based on obtaining a high degree of tissue transparency and matching of the sample refractive index (RI) to that of the imaging media to remove light scattering (54). Most tissues are comprised of ~80% water (RI=1.33), 10% proteins (RI>1.44), and 10% lipids (RI>1.45) (55). Methods are broadly based on physical or chemical strategies with organic solvents or aqueous solutions used for the latter. Several excellent reviews are available that detail each optical clearing method advantages and disadvantages (45, 56–59). Early optical clearing methods used organic chemicals [e.g., benzyl alcohol–methyl salicylate, benzyl alcohol–benzyl benzoate (BABB), and solvents used in 3-D imaging of solvent-cleared organs (3DISCO)] (27). Generally these methods achieve high transparency within a few days by removing lipids and homogenizing refractive indices (RIs) of the samples, and they are compatible with whole-mount immunolabeling (48, 60). Solvent based clearing methods may use toxic chemicals and steps should be performed using a fume hood and suitable personal protective equipment. Recent methods have been named with acronyms such as immunolabeling-enabled imaging of solvent-cleared organs (iDISCO) (60), clear, unobstructed brain/body imaging cocktails and computational analysis (CUBIC) (17, 27, 61, 62), clear lipid-exchanged acrylamide-hybridized rigid imaging/immunostaining/*in situ* hybridization-compatible tissue hydrogel (CLARITY), and passive CLARITY technique (PACT) (38, 63–65).

**Table 1:** Optical clearing references in human and mouse pancreas.

Species	First Author	Year	Journal	Title	Method	Reference
Mouse	Kim	2010	JoVE	In situ quantification of pancreatic beta-cell mass in mice	Sucrose	(18)
Mouse	Fu	2010	Gastroenterology	At the movies: 3-dimensional technology and gastrointestinal histology	FocusClear	(19)
Mouse	Fu	2010	Journal of Biomedical Optics	Three-dimensional optical method for integrated visualization of mouse islet microstructure and vascular network with subcellular-level resolution	FocusClear	(20)
Rat	Li	2010	J Cell Science	Activation of pancreatic-duct-derived progenitor cells during pancreas regeneration in adult rats	BABB	(21)
Mouse	Chiu	2012	Diabetologia	3-D imaging and illustration of the perfusive mouse islet sympathetic innervation and its remodeling in injury	FocusClear	(22)
Mouse	Tang	2013	Diabetologia	Plasticity of Schwann cells and pericytes in response to islet injury in mice	FocusClear	(23)
Mouse	Juang	2014	AJP	Three-dimensional islet graft histology: panoramic imaging of neural plasticity in sympathetic reinnervation of transplanted islets under the kidney capsule	FocusClear	(24)
Mouse	Tang	2014	Diabetes, Obesity and Metabolism	Imaging of the islet neural network	FocusClear	(25)
Mouse	Lee	2014	BMC Developmental Biology	Improved application of the electrophoretic tissue clearing technology, CLARITY, to intact solid organs including brain, pancreas, liver, kidney, lung, and intestine	CLARITY	(26)
Mouse	Susako	2015	Nature Protocols	Advanced CUBIC protocols for whole-brain and whole-body clearing and imaging	CUBIC	(27)
Mouse	Juang	2015	EBioMedicine	3-D imaging reveals participation of donor islet Schwann cells and pericytes in islet transplantation and graft neurovascular regeneration	FocusClear/RapiClear	(28)
Human	Treweek	2015	Nature Protocols	Whole-body tissue stabilization and selective extractions *via* tissue-hydrogel hybrids for high-resolution intact circuit mapping and phenotyping	PACT	(29)
Mouse	Chien	2016	International Journal of Obesity	3-D imaging of islets in obesity: formation of the islet-duct complex and neurovascular remodeling in young hyperphagic mice	RapiClear	(30)
Mouse	Lin	2016	AJP	PanIN-associated pericyte, glial, and islet remodeling in mice revealed by 3-D pancreatic duct lesion histology	RapiClear	(31)
Mouse	Simon	2017	J Autoimmunity	Inhibition of effector antigen-specific T cells by intradermal administration of heme oxygenase-1 inducers	3DISCO	(32)
Mouse	Vlahos	2017	PNAS	Modular tissue engineering for the vascularization of subcutaneously transplanted pancreatic islets	CLARITY	(33)
Mouse	Wong	2017	Current Protocols Cell Biology	Simple and Rapid Tissue Clearing Method for Three-Dimensional Histology of the Pancreas	CLARITY	(34)
Mouse	Yamamoto	2017	Nat Comm	Neuronal signals regulate obesity induced β-cell proliferation by FoxM1 dependent mechanism	CUBIC	(35)
Mouse	Pauerstein	2017	Development	A radial axis defined by semaphorin-to-neuropilin signaling controls pancreatic islet morphogenesis	CLARITY	(36)
Mouse	Chen	2017	Scientific Reports	UbasM: An effective balanced optical clearing method for intact biomedical imaging	UbasM	(37)
Mouse, Human	Hsueh	2017	Nature Protocols	Pathways to clinical CLARITY	CLARITY	(38)
Mouse	Tang	2018	Diabetologia	Pancreatic neuro-insular network in young mice revealed by 3-D panoramic histology	RapiClear	(39)
Mouse	Nishimura	2018	Islets	Optical clearing of the pancreas for visualization of mature b-cells and vessels in mice	Sca/eS	(40)
Human	Noë	2018	American Journal of Pathology	Immunolabeling of Cleared Human Pancreata Provides Insights into Three-Dimensional Pancreatic Anatomy and Pathology	iDISCO	(41)
Human	Tang	2018	Diabetologia	Human pancreatic neuro-insular network in health and fatty infiltration	RapiClear	(42)
Human	Tang	2018	Current Diabetes Reports	The role of accessory cells in islet homeostasis	RapiClear, PACT	(14)
Human	Fowler	2018	Endocrinology	Three-Dimensional Analysis of the Human Pancreas	T3	(43)
Human	Butterworth	2018	JoVE	High resolution 3D imaging of the Human Pancreas Neuro-insular network	PACT	(15)
Human, Mouse	Shen	2019	EBioMedicine	Lymphatic vessel remodeling and invasion in pancreatic cancer progression	RapiClear	(44)
Human	Chien	2019	AJP	Human pancreatic afferent and efferent nerves: mapping and 3-D illustration of exocrine, endocrine, and adipose innervation	RapiClear	(13)
Human	Dybala	2019	Diabetes	Heterogeneity Human Pancreatic Islet	T3	(2)
Human	Hong	2019	Advances in Anatomic Pathology	A “Clearer” View of Pancreatic Pathology: A Review of Tissue Clearing and Advanced Microscopy Techniques	iDISCO	(45)
Mouse	Tokumoto	2020	Diabetes	Generation and Characterization of a Novel Mouse Model That Allows Spatiotemporal Quantification of Pancreatic β-Cell Proliferation	CUBIC	(46)
Mouse	Hahn	2020	Communications Biology	Topologically selective islet vulnerability and self-sustained downregulation of markers for β-cell maturity in streptozotocin-induced diabetes	BABB	(47)
Mouse	Maldonado	2020	Stem Cells Tissue Repair	Painting the Pancreas in Three Dimensions: Whole-Mount Immunofluorescence Method	BABB	(48)
(49)Human, Mouse	Alvarsson	2020	Science Advances	3D atlas of the dynamic and regional variation of pancreatic innervation in diabetes	iDISCO (modified), ECi	(12)
Human	Hong	2020	Mod Pathology	Three-dimensional visualization of cleared human pancreas cancer reveals that sustained epithelial-to-mesenchymal transition is not required for venous invasion	iDISCO	(50)
Human	Heuckeroth	2020	Gastroenterology	Robust, 3-Dimensional Visualization of Human Colon Enteric Nervous System Without Tissue Sectioning	BABB	(51)
Mouse, Human	Chen	2021	EMBO	Decreased blood vessel density and endothelial cell subset dynamics during ageing of the endocrine system	PEGASOS	(52)
Human	Campbell-Thompson	2021	Scientific Reports	Islet Sympathetic Innervation and Islet Neuropathology in Patients with Type 1 Diabetes	iDISCO, PACT	(53)

The original CLARITY manuscript by Chung et al. (65) described four key steps: (1) hydrogel tissue embedding using a ratio of 4% acrylamide monomer to 0.05% bis-acrylamide followed by polymerization; (2) clearing secondary to lipid removal using 4% sodium dodecyl sulfate (SDS) detergent buffer within a custom built electrophoretic tissue clearing system (ETC); (3) immunostaining; and (4) tissue RI matching and imaging. Several modifications were subsequently published in favor of “passive clearing” without the use of ETC to avoid oxidative tissue artifacts with several variations in the amount of paraformaldehyde (0-4%), acrylamide monomer (1-4%) and bis-acrylamide in the hydrogel mixture depending on target organ (63, 66, 67).

Despite their differences in chemical and/or optical properties, the organic solvent, aqueous reagent, and electrophoresis-assisted clearing methods all extend our view of neurovascular networks (>>100 µm) with 3-D microscopy compared with the images acquired from classic IHC and H&E histology (3-5 µm in thickness). To apply tissue clearing, the key question is whether a specific clearing technique changes the sample chemical and/or cellular environment that causes artifacts in signal detection. For example, in CLARITY, the use of sodium dodecyl sulfate (SDS) treatment and electrophoresis to remove cellular membranes is likely to disturb membrane receptor proteins. Thus, studies of the nerve-receptor association in space will be better accepted if a passive aqueous-based clearing method (e.g., sugar reagent) is employed compared with CLARITY. Likewise, leukocytes and their vascular receptor association in space will be better examined in an aqueous environment, because disturbing the membranes for 3-D imaging may create false negative results in signal detection. However, we need to stress that the false negative result may also come from scattering in deep-tissue imaging. Thus, adding a positive control in the specimen (e.g., nuclear staining) can help investigators monitor the resolving power (e.g., resolving two adjacent nuclei in an islet) across the optical depth in deep-tissue pancreatic and islet imaging.

Volumetric microscopy has also paralleled optical clearing methods with advancements in confocal, multiphoton and lightsheet microscopes. Advantages of the lightsheet microscopes are faster scanning speeds, reduced photobleaching, and good resolution at high tissue penetration depths. When access to lightsheet microscopes is not feasible, confocal microscopy provides an excellent alternative with single-cell resolution. While any type of fluorescent microscope can be used to image optically-cleared tissue, the microscope imaging chamber must be able to accommodate the size of the sample and the stage configuration and imaging depth are dependent on the working distance of the objective. Most images can be obtained with regular air objectives between ×2 and ×20 magnifications. Specialized objectives for optically cleared samples over larger distances are available from multiple vendors such as Olympus and Zeiss. Users of optical clearing methods and advanced microscopy need also consider data storage requirements for both image acquisition and analysis. Additional storage space for the imaging microscope is required as well as additional memory and processing speed for image analysis workstations. The opensource software Fiji/ImageJ can be used for 2-D stitching and basic 3-D adjustments (68). Zeiss Zen software will also provide stitching and maximum intensity projections. Commercial software packages are available for advanced 3-D volume rendering and reconstruction such as Neurolucida360/Vesselucida (MBF), Arivis (FEI), and Imaris (Bitplane, Concord MA). Consideration of sample size and existing microscopes and image analysis software may thus dictate which optical clearing method is most suitable for a given laboratory. For those without sufficient local resources, the growing popularity of optical clearing methods generated several commercial sources services for clearing, microscopy, and analysis services (Visikol, ClearLight, LifeCanvas).

## Challenges

Challenges related to optical clearing are relatively few. Acquisition of high-quality samples is important as for any down-stream application based on fixed samples. Fixation with 4% paraformaldehyde or 10% formalin is commonly employed in many laboratories. Cardiac perfusion is recommended for rodent studies in most part to remove red blood cells and their inherent high autofluorescence. However, immersion fixation is a reasonable alternative for rodents and the only method available for human biosamples. As for traditional immunolocalization, the duration of fixation is ideally kept to a minimum to avoid over-fixation of tissue antigens. Many primary antibodies utilized for formalin-fixed paraffin embedded samples work well for optical clearing and those tested in our laboratory are provided as a reference (**Table 2**). A pre-testing step is advised for new primary antibodies and can be accomplished with fixed frozen thick sections (40µm) utilizing similar conditions as those for permeabilization before immunostaining and optical clearing. Wide applicability of primary antibodies remains an issue, particularly for immune markers. While some methods have successfully employed preconjugated primary antibodies (43), the majority of optical clearing methods employ standard rounds of primary antibodies followed by secondary antibodies to promote antibody penetration. A reported benefit of CLARITY was the ability to reiteratively strip antibodies and reprobe a sample several times (65). In practice, we have been unsuccessful in fully stripping samples from human pancreas cleared using CLARITY and also found limited antigenicity and/or diffusion of subsequent primary antibodies (Campbell-Thompson, unpublished results). As such, our more recent studies employ single clearing methods with multiplex immunostaining on 500µm sections rather than several mm sized pieces to extend use of a given sample, particularly those from rare organ donors with diabetes.

**Table 2:** Primary antibodies for optical clearing.

Antigen	Cell type	Host	Vendor	Cat. #	Dilution	Comments
**Endocrine Markers**						
Glucagon	Alpha-cells	Mouse	BD Biosciences	565891	1:50	Worked
Glucagon	Alpha-cells	Rabbit	Cell Signaling	2760S	1:200	Did not work
Glucagon	Alpha-cells	Mouse	Abcam	ab10988	1:200	Worked
Insulin	Beta-cells	Guinea Pig	DAKO	A0564	1:200	Worked
Secretogranin 3	Neuroendocrine cells	Rabbit	Sigma	HPA006880	1:200	Worked
Somatostatin	Delta-cells	Goat	Santa Cruz	sc-7819	1:500	Worked
**Neural Markers**						
GFAP	Glial cells	Rabbit	DAKO	Z0334	1:200	Worked
NCAM (CD56)	Pan-neural	Mouse	DAKO	M730429-2 (also FITC- conjugate)	1:50	Did not work (both)
Peripherin	Pan-neural	Rabbit	EnCor	RPCA-Peri	1:200	Worked
PGP9.5/UCHL1	Pan-neural	Rabbit	DAKO	Z5116	1:50	Did not work
PGP9.5/UCHL1	Pan-neural	Chicken	EnCor	CPCA-UCHL1	1:100	Worked
PGP9.5/UCHL1	Pan-neural	Rabbit	Abcam	ab108986	1:200	Worked
β-Tubulin	Pan-neural	Mouse	EnCor	MCA-4E4	1:100	Worked
Substance P	Sensory nerves	Rat	BioRad	8450-0505	1:200	Worked
Tyrosine Hydroxylase	Sympathetic neurons	Rabbit	Millipore	AB152	1:200	Worked
Tyrosine Hydroxylase	Sympathetic neurons	Chicken	Abcam	Ab76442	1:50	Worked, weak staining
Vasoactive Intestinal Peptide	Autonomic neurons	Rabbit	Immunostar	20077	1:200	Worked
Vesicular acetylcholine transporter	Cholinergic neurons	Rabbit	Synaptic Systems	139103	1:200	Worked
**Vascular Markers**						
CD31 (PECAM)	Endothelial cells	Rabbit	Abcam	Ab28364	1:30	Worked
CD31 (PECAM)	Endothelial cells	Mouse	ThermoFisher	MS-353-S1	1:50	Worked, weak staining
CD34	Endothelial cells	Mouse	ThermoFisher	MA1-10202	1:50	Did not work
Collagen IV	Basal lamina (blood vessels)	Mouse	Abcam	ab6311	1:200	Did not work
Collagen IV	Basal lamina (blood vessels)	Rabbit	Abcam	ab6586	1:200	Worked
Smooth muscle actin	Smooth muscle (arteries)	Mouse	Sigma	A5228; C6198 (Cy5)	1:200; 1:200	Worked; Conjugated better

Primary antibodies tested for immunolocalization in human pancreas samples cleared by passive CLARITY (PACT) are shown by major headings for endocrine markers, neural markers, and vasculature. Comments include whether successful immunostaining was achieved. These antibodies are expected to work using similar clearing methods and may also work in other species as indicated by the vendor or literature.

Equipment utilized in optical clearing is generally found in any modern molecular pathology laboratory and include access to fume hoods, refrigeration or ovens, rocker plates, and other ancillary small equipment for immunostaining and clearing steps. Microscope and image analysis software are two aspects to be considered before conducting clearing studies as this will influence sample size and numbers of channels for multiplex staining and analysis. As costs for multiphoton and lightsheet microscopes decrease, access to these microscopes will increase whether through institutional shared resources, subcontract, or collaboration. Image analysis expertise is also limited and laboratory staff become proficient in the software available to them. Expense of commercial 3-D software analysis programs is high. Finally, the greatest hurdle may be the large image file sizes achieved by these methods. Here too, limiting the number of antigens needs to be balanced with the rarity of the sample since reiterative staining is difficult.

## Optical Clearing Examples

This introduction on optical clearing and imaging provides a basic starting point for studies on human and mouse pancreas in health and diabetes. We will now show examples of application of different clearing methods to demonstrate the versatility of optical clearing in human and mouse pancreas for determination of normal states and changes found with diabetes.

## Sample Processing

Human pancreata not suitable for clinical purposes were collected from nondiabetic, brain-dead organ donors after written informed consent from legal representative or next of kin and were processed by the Network for Pancreatic Organ donors with Diabetes (nPOD) program at the University of Florida (UF) Diabetes Institute using methods previously reported (69). The nPOD samples used in this specific study were approved as nonhuman by the UF IRB (IRB201902530). Collection and use of human pancreatectomy specimens were approved by the Institutional Review Board of National Taiwan University Hospital (201703131RIND). All UF animal studies were conducted using published guidelines and regulations of the National Institutes of Health for the care and use of laboratory animals. The protocol was approved by the UF Institutional Animal Care and Use Committee (IACUC 202009976). All animal studies conducted at the National Tsing Hua University were reviewed and approved by the institutional animal use review board.

### Pancreatic Islet Schwann Cells

**Figure 1:**
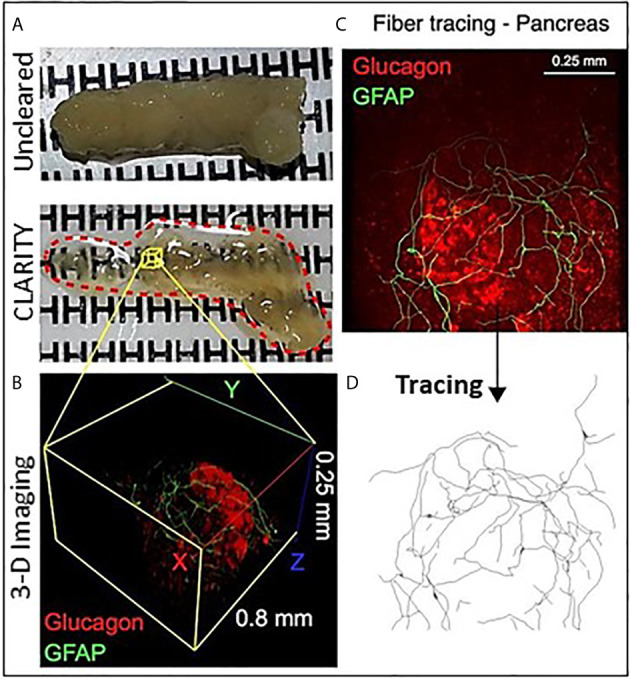
Optical clearing of human pancreas by PACT and iDISCO. **(A)** Fixed pancreas sample from a control donor before and after clearing using passive CLARITY (PACT) showing the degree of sample transparency achieved with this method. **(B)** Representative example of 2-photon imaging for a 0.8 mm x 0.8 mm x 0.25 mm region (X, Y, Z axes) containing an islet immunostained for glucagon (red) and glial fibrillary acidic protein (GFAP, green). **(C)** GFAP-stained Schwann cells overlay an islet and cell projects were analyzed using the ImageJ neurite tracer program. **(D)** The traced Schwann cells are shown by Neurite tracer skeleton diagram.

**Figure 2:**
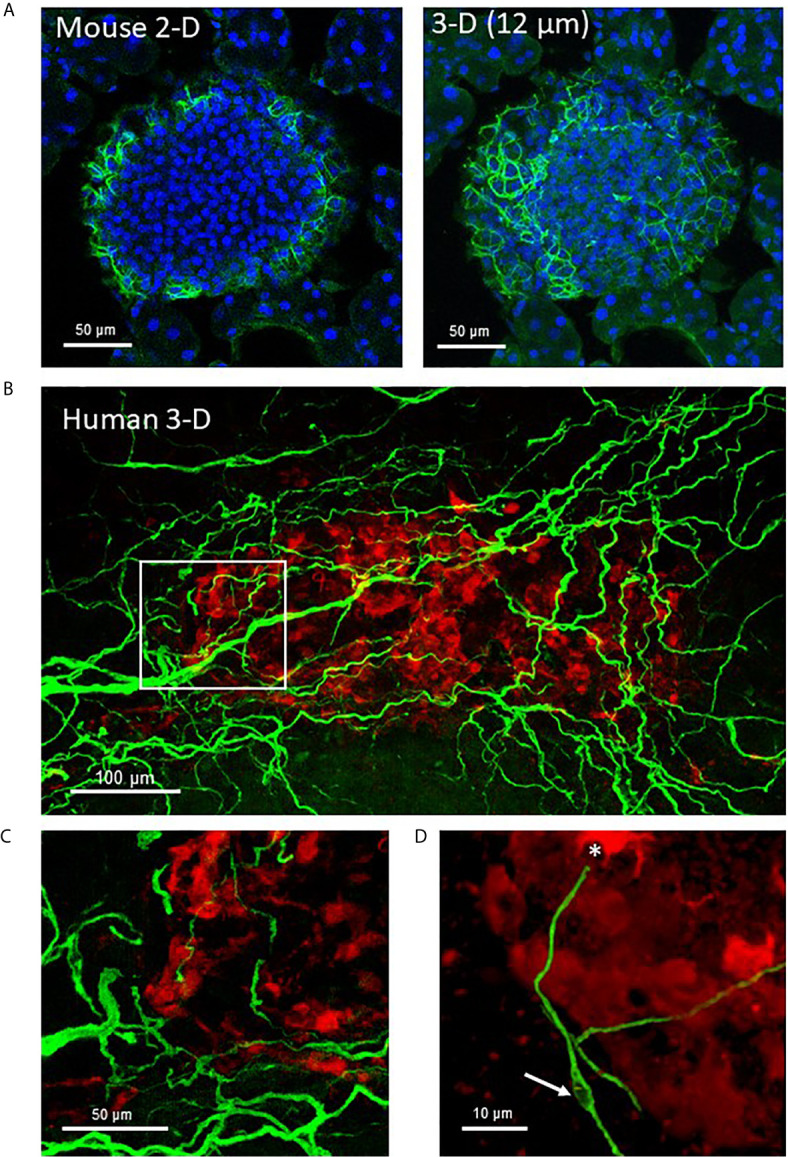
Mouse and human islet Schwann cells. **(A)** A fixed frozen section (40µm) from a C57BL/6 mouse pancreas was stained for GFAP (green) using whole mount staining. A single 2-D slice (7^th^ of 13 slices) and the 3-D maximum intensity projection (MIP, 12 µm stack) are shown to demonstrate the increase in cellular information obtained with a z-stack. **(B)** A human control pancreas sample (~1 mm^3^) was cleared by iDISCO and immunolabeling with glucagon (red) and GFAP (green) before confocal 3-D imaging (maximum intensity projection 50 µm). **(C)** The region identified by white box in **(A)** shows Schwann cells at the periphery of the islet that extended along nerves to islet interiors traveling along afferent vessels. **(D)** A single Schwann cell shows a clear nuclear region (white arrow) and numerous extensions with a termination at an alpha-cell (asterisk). See also **Supplementary Video 1** for **(A)**.

Passive CLARITY (PACT) provides for good tissue transparency and multiplex immunolabeling is quite feasible with image analysis such as using the open software Neurite tracer program in ImageJ (**Figure 1**) **(** 15). Schwann cells are the peripheral counterpart to central nervous system oligodendrocytes and provide support to both myelinated and unmyelinated axons of motor and sensory neurons (70, 71). In addition to providing physical support, nonmyelinating Schwann cells are essential for maintenance and regeneration of damaged axons by production of neurotrophins and acting as “first responders” to injury (72, 73). Unlike the dense mesh-like network formed by islet Schwann cells in mouse islets, Schwann cells provide support for autonomic nerves in human islets in a loose formation (**Figure 2**) **(** 70). Schwann cells are also of particular interest in T1D as they have a role in antigen presentation, interact with the complement system, and secrete factors involved in immune interactions (74, 75) and animal studies report reactive Schwann cells in diabetes and islet injury (23, 76–78). Furthermore, glial fibrillary acidic protein (GFAP) is expressed in peri-islet Schwann cells and is reported to be an autoantigen for T1D with potential use as a biomarker (79).

### Pancreatic Ganglia and Neuroinsular Complexes

**Figure 3:**
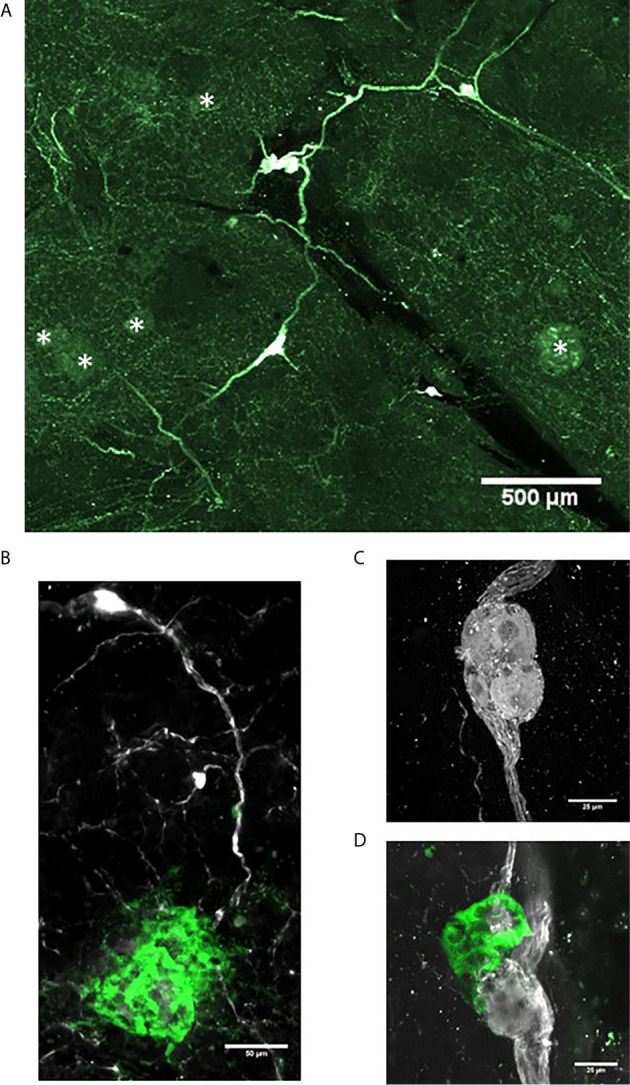
Intrapancreatic ganglia and neuroinsular complexes in adult human pancreas. Pancreas samples were studied in control donors using passive CLARITY (PACT) and immunostaining with primary antibodies for PGP9.5 (white) and GFAP (green) to delineate nerve fibers and supporting Schwann cells, respectively (**Table 2**). PGP9.5 also stained islet endocrine cells although with much less intensity (asterisks). Intrapancreatic ganglia represent post-ganglionic neurons and fibers of the parasympathetic efferent system and 3-D imaging shows how ganglia are interconnected **(A)** and also extend to islets **(B)**. **(C)** Intrapancreatic ganglia contained varying numbers of neurons and small collections of neurons were also found widely scattered with efferent and afferent axons in both interlobular and intralobular regions. **(D)** Imaging for PGP9.5 and islet alpha-cells (GCG, green) demonstrate close association of clustered alpha-cells with neurons at a small ganglion, also known as a neuro-insular complex II. Scale bars: 500µm **(A)**, 50µm **(B)**, 25 µm **(C, D)**. See also **Supplementary Video 2** for **(D)**.

Intrapancreatic ganglia represent the post-ganglionic neurons of the parasympathetic efferent network (80). They are widely distributed and in relatively low density throughout the human and rodent pancreas and thus optical clearing and 3-D imaging provides a greater opportunity to detect these ganglia (13). At low magnifications, interconnections of intrapancreatic ganglia and to islets are visualized in a human pancreas cleared using PACT (15) (**Figures 3A, B**). Such interconnections likely contribute to the synchronization of islet hormone secretions particularly during the cephalic phase of digestion (81). The intrapancreatic ganglia vary in numbers of neurons and also observed are small clusters of neurons (**Figure 3C**) or small clusters of neurons with islet β-cells and α-cells can also be observed, so-called neuroinsular complex type II (**Figure 3D**) (82). These type II structures have not been previously reported in adult human pancreas and were found only in fetal pancreas. The lack of detection in adults could be due to limitations of 2-D microscopy in finding small structures compared to 3-D microscopy as demonstrated here (83).

### Pancreatic Vasculature

**Figure 4:**
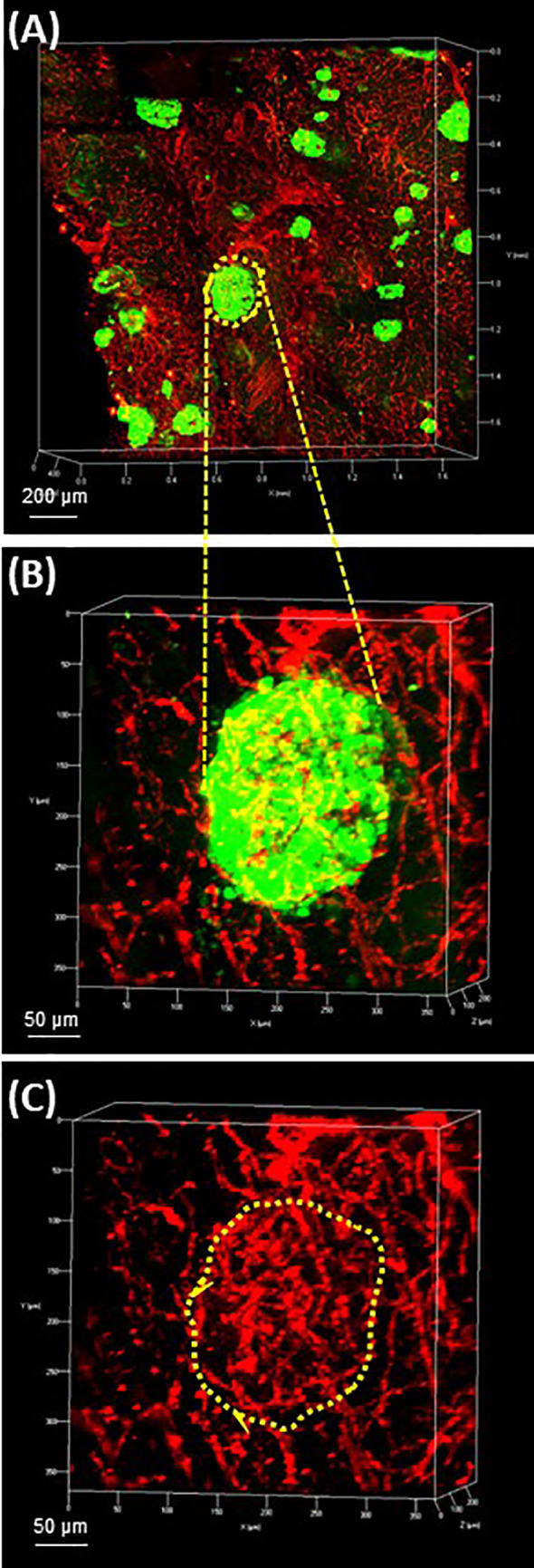
Human pancreas vasculature. 3-D extended projection of human pancreas exocrine and endocrine vasculature are shown with X, Y and Z axes in mm (Scale bar 200 µm). **(A)** The extensive nature of the human pancreas vascular system is demonstrated by immunolabeling with monoclonal anti-CD31 (red) and islets are shown stained with monoclonal anti-glucagon (green) antibodies. **(B)** An islet identified by yellow-dotted line in **(A)** is shown at higher resolution in **(B)** (Scale bar 50 µm). **(C)** The islet microvasculature is shown without the glucagon overlay (Scale bar 50 µm). See also **Supplementary Video 3** for **(A–C)**.

The pancreatic vasculature in health and diabetes has been studied with newer studies showing detection of the Sars-CoV-2 receptor, ACE2, in pancreatic micovasculature, rather than islet endocrine cells, adding additional importance to understanding factors regulating islet blood flow in health and diabetes (84–87). The use of optical clearing provides an unprecedented opportunity to better examine structural-functional relationships of the islet microvasculature in the context of islet heterogeneity and inter-relationship to the surrounding acinar cells. Studies performed in rodents can be achieved by perfusion with fluorescent compounds including lectins or conjugated primary antibodies such as CD31. For human samples, the vasculature can be readily labeled using CD31 or CD34 followed by multiplex immunofluorescence with islet endocrine cell markers and the high vascular density can be appreciated throughout 3-D microscopy (**Figure 4**) (53).

### Pancreatic Acinar Ductal Metaplasia

**Figure 5:**
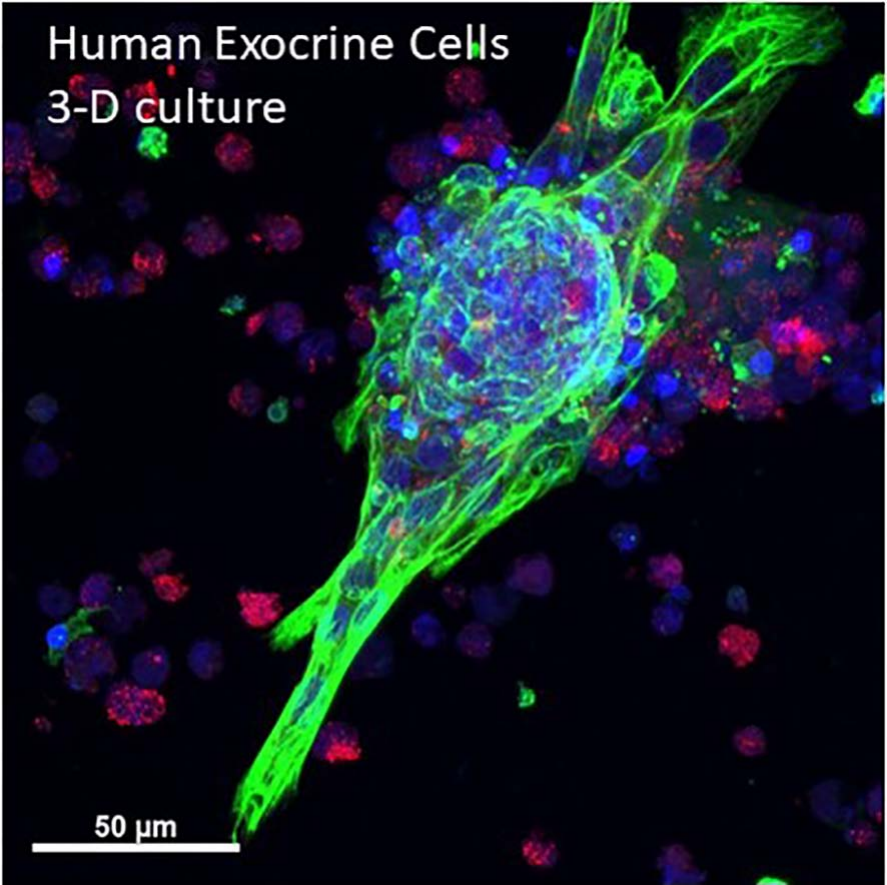
3-D projection human primary pancreas exocrine cells in culture. Isolated human exocrine cells were obtained from a pancreas donor non-islet fractions and following filtration to remove islets and clumps, exocrine cells were plated in 1:1 DMEM:F12 and Matrigel and grown for 6 days. A maximum projection image shows ductal cells (cytokeratin 19, green), acinar cells (amylase, red) and nuclei (blue). Cells were kindly provided by Dr. Thomas Schmittgen, College of Pharmacy, University of Florida. See also **Supplementary Video 4** for entire 3-D z-stack (12 µm).

Pancreatic cancer is one of the deadliest tumors and seminal studies showed that early lesions likely arise from acinar-ductal metaplasia forming so-called pancreatic intraductal neoplasia (PanIN) (88). Optical clearing studies in human and mice have shown characteristics of ductal lesions through 3-D imaging (31, 50). Cell culture models of human pancreatic cancer can also benefit from use of optical clearing and 3-D microscopy. Single cell details are apparent in an *in vitro* human primary acinar culture model showing duct formation following optical clearing using HISTO-M, a commercial product similar to iDISCO clearing (**Figure 5**) (Visikol) (T. Schmittgen, personal communication) (89).

### Pancreatic Fatty Infiltration

Unlike the fatty liver, in which lipid droplets accumulate in the cytoplasm in the hepatocytes, pancreatic fatty infiltration involves the fat cells (adipocytes) ectopically developing and accumulating in and around the pancreatic lobules alongside the exocrine and endocrine tissues. The fat content in the pancreas increases with age (90) and is detectable as early as in adolescence (91), and the degree is linked with obesity (92–94). In the progression from obesity to type 2 diabetes, the state of hyperinsulinemia is likely to accelerate the pancreatic fat accumulation due to the organ’s high insulin concentration. Insulin is a potent factor to induce adipogenesis, in which preadipocytes (e.g., fibroblasts and myofibroblasts in the pancreatic stroma) differentiate into adipocytes (95, 96), and stimulate the proliferation of adipocytes (97). Thus, it is not surprising that multiple studies documented the correlation between type 2 diabetes and pancreatic fats and implicated the negative influence of these fats on the islet microenvironment and function (98–100).

Clinically, magnetic resonance imaging (MRI) and computed tomography (CT) are the preferred imaging modalities to detect and quantify the pancreatic fats (90–92, 94, 98–102). While these two methods provide valuable *in vivo* information for cross-sectional or longitudinal studies, they cannot resolve the cellular structures of fats and the pancreatic exocrine and endocrine tissues. At the cellular level, the classic microtome-based histology with H&E staining can identify the adipocytes and their association with blood vessels, acini, ducts, and islets with µm-level resolution. However, due to the hydrophobicity of fats and their weak mechanical connection with the pancreatic lobules, both microtome slicing and dewaxing (xylene wash) of the paraffin-embedded pancreas may create artifacts on the locations of adipocytes, affecting the analysis of the peri- and/or intra-lobular adipocyte association.

**Figure 6:**
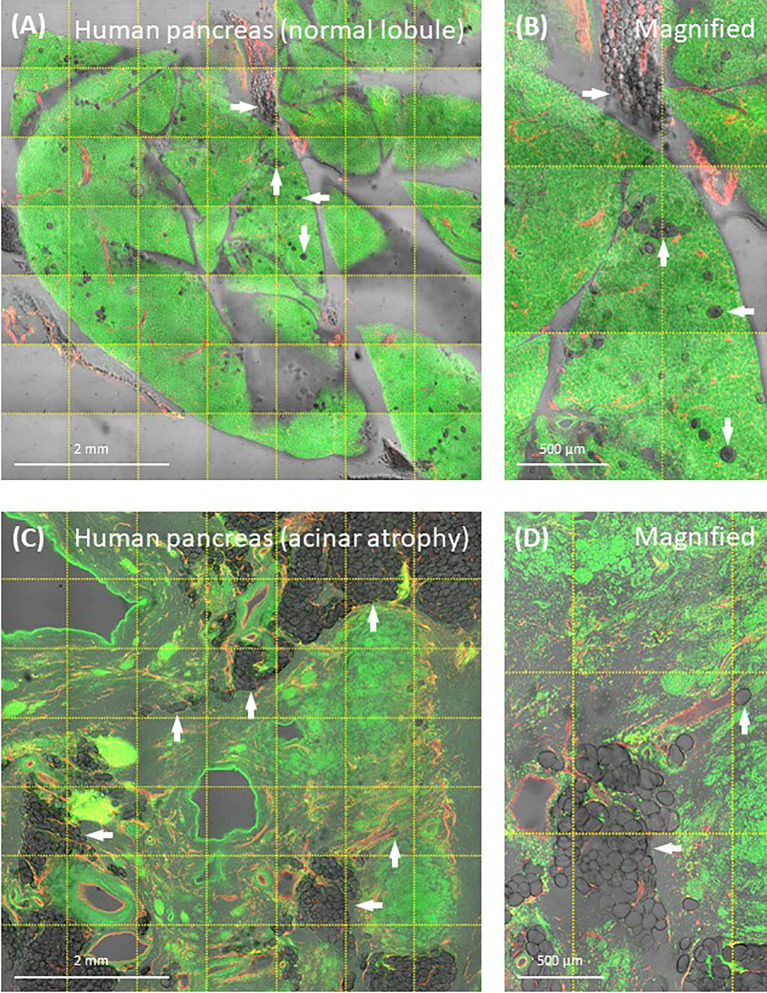
Human pancreatic fatty infiltration. Images were derived from tile scanning of optically cleared pancreatic specimens. **(A, B)** Normal lobule of human pancreas. Adipocytes are clearly seen around the blood vessel and inside the lobule (magnified, arrows). Green, nuclear staining; red, CD31. **(C, D)** Acinar atrophy of diseased lobule. This view was acquired 2-cm distal to the pancreatic ductal adenocarcinoma. Overlay of transmitted light and fluorescence signals identifies the fatty infiltration.

Modern 3-D histology with aqueous-based optical clearing alleviates the abovementioned technical concern by maintaining the native hydrophilicity of the tissue and avoiding the microtome slicing in sample preparation (42). This is particularly important in examination of the type 2 diabetic pancreas and the surgical biopsy of pancreatic cancer [patient may have developed type 3c diabetes (103, 104)]. In both situations, investigators will likely encounter moderate-to-severe fatty infiltration, in which adipocytes generally or locally become a component of the pancreas (**Figure 6**). To investigate the pancreas in this condition, we advise careful comparison between the modern 3-D and the classic 2-D tissue images to confirm the adipocytes in and around the remodeled pancreatic lobules to avoid misrepresentation of the disease condition.

### Pancreatic Lymphatic Network

The lymphatic drainage of pancreas is achieved by an intricate network of lymphatic vessels and nodes, in which the immune cells reside. The open-ended lymphatic network collects the interstitial fluids for water and lipid absorption and recycling (which balances the tissue osmatic pressure) and for immune surveillance (105). When pancreatic injury or disease occurs, the lymphatic system plays a central role in reaction to the exocrine [pancreatitis (44, 106)] and endocrine tissue inflammation. For example, in the nonobese diabetic (NOD) mice, the progression of type 1 diabetes features the migration of T lymphocytes from the circulatory system to the islet, attacking the β-cells (107, 108). In the process, the microtome-based 2-D histology has been used to evaluate the degree of islet inflammation, in which early, moderate, and severe insulitis are assigned to evaluate the progression of the disease (109). However, due to the dispersed nature of lymphatic vessels, the classic 2-D histology cannot provide a global assessment of lymphatic endothelial remodeling in response to insulitis. To understand the associated lymphatic and immune response to insulitis, panoramic and in-depth imaging of the pancreas is needed to characterize the lymphatic network and T-cell migration in a global and integrated fashion.

**Figure 7:**
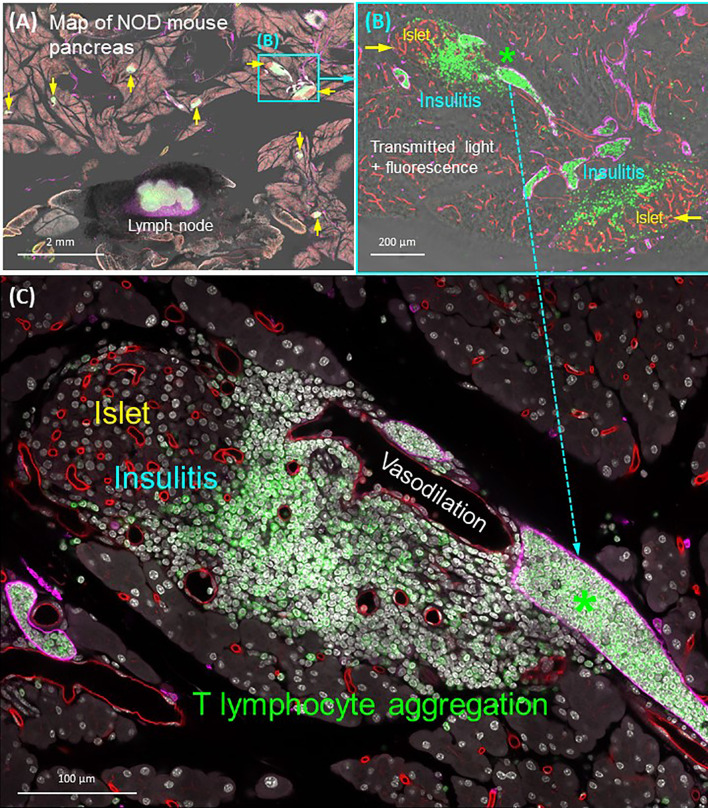
Panoramic and high-resolution images of optically cleared NOD mouse pancreas with insulitis. **(A)** Map of 8-week NOD mouse pancreas. Overlay of transmitted light and fluorescence signals identifies the Lyve1^+^ lymph node (filled with CD3^+^ T lymphocytes and surrounded by fats) and locations of insulitic islets (yellow arrows). Two islets (cyan box) are magnified in **(B)**. **(B)** Islets with insulitis are shown with blood vessels (red), lymphatic vessels (magenta), and nuclei (white). CD3^+^ T lymphocytes are identified around the islets and congregated in the lymphatic vessels (asterisk; vascular compartment vs. extravascular domain). This feature is further magnified in **(C)**. **(C)** Peri-islet aggregation of T lymphocytes and their vascular association shown as well as the peri-islet vasodilation and lymphocytic infiltration. See also **Supplementary Video 5** for **(B)**.

As can be seen in **Figure 7**, the optically cleared NOD mouse pancreas provides an experimental setting to investigate the lymphatic and T-lymphocyte association in insulitis. Using the panoramic image (**Figure 7A**), we can detect the CD3^+^ T lymphocytes in the lymph node (positive control) and around the islets. The latter provides a clear target for examination of the islet under immune attack, featuring vasodilation and the packing of T lymphocytes in the lymphatic vessels (**Figures 7B, C**). Overall, the quadruple signals of tissue microstructure (nuclear staining), vasculature (blood and lymphatic vessels), and CD3^+^ T lymphocytes in the transparent pancreas provide an optimized condition to visualize the islet vascular remodeling and immune attack in insulitis. They also demonstrate the different scales of tissue information (from interlobular to subcellular features of islets under immune attack) that can be acquired from optically cleared pancreas samples to investigate experimental insulitis.

## Conclusions

Both standard and modified optical clearing methods are well suited for studies of 3-D structure-function relationships for human and mouse pancreas and use readily available chemicals and imaging equipment. These methods are particularly advantageous for studies of diabetes due to known islet heterogeneity requiring examination of numerous islets and pancreas regions. Optical clearing methods can also be used in investigations of pancreatic cancer using patient or rodent samples or *in vitro* experiments examining acinar-ductal metaplasia. Advances in understanding failure of islet beta-cells in diabetes requires a wholistic examination of islets in their native environment as afforded by optical clearing and new findings are anticipated related to the role of the nervous, immune, and vascular systems in beta-cell biology from such studies.

## Author Contributions

MC-T and S-CT designed the studies, performed experiments, prepared figures, and edited and revised the manuscript. All authors contributed to the article and approved the submitted version.

## Funding

Funding provided by NIH 1R01DK122160, UC4 DK104155, U54 DK127823 and OT2 OD023861, Helmsley Charitable Trust 2015PG-T1D052 and JDRF 47-2014-1 and 2-SRA-2019-697-S-B to MC-T and Taiwan National Health Research Institutes (NHRI-EX109-10922EI) and Ministry of Science and Technology (MOST 108-2314-B-007-006-MY2) to S-CT.

## Conflict of Interest

The authors declare that the research was conducted in the absence of any commercial or financial relationships that could be construed as a potential conflict of interest.
