# Supplementary material for: Pancreas Optical Clearing and 3-D Microscopy in Health and Diabetes
Source: Front Endocrinol (Lausanne). 2021 Apr 26;12:644826. doi: 10.3389/fendo.2021.644826 (PMC8108133; doi:10.3389/fendo.2021.644826)
Supplement: Supplementary file 1 [file DataSheet_1.docx]

Pancreas Optical Clearing and 3-D Microscopy

in Health and Diabetes

Martha Campbell-Thompson^1^, Shiue-Cheng Tang^2^

^1^Department of Pathology, Immunology and Laboratory Medicine, 1395 Center Drive, College of Medicine, University of Florida, Gainesville FL, 32610, USA

^2^Department of Medical Science and Institute of Biotechnology, National Tsing Hua University, Hsinchu 30013, Taiwan

*** Correspondence:**Martha Campbell-Thompson
[mct@ufl.edu](mailto:mct@ufl.edu)

**Shiue-Cheng Tang**

sctang@life.nthu.edu.tw

**This file includes:**

Legends for videos 1-5

**Other Supplementary Material for this manuscript includes the following:**

Videos 1-5

**LEGENDS TO SUPPLEMENTARY VIDEOS**

**Supplementary Video 1. Mouse and Human Schwann cells.** A fixed frozen (40 µm) section of pancreas from a C57BL/6 female mouse was stained with GFAP (green) and a nuclear marker (blue) by whole mount staining. Enhancement of Schwann cell distribution can be visualized by 3-D microscope. The video shows a confocal image z-stack imaged using a 40x/0.95 objective on a Zeiss LSM 710 (12 slices imaged at 1.0 µm z-steps. Scale bar 50 µm.

**Supplementary Video 2. Neuroinsular complex in adult human pancreas.** Pancreas sample from a control donor was cleared using PACT and immunostained with primary antibodies for PGP9.5 (white) and Glucagon (green) to delineate nerve fibers and alpha-cells, respectively. The video shows a confocal image stack imaged using a 40x/0.95 objective on a Zeiss LSM 710 (42 slices imaged at 0.97 µm each, total 39.8 µm). The cluster of alpha-cells appears immediately adjacent to underlying neurons and axons. Scale bar 20 µm.

**Supplementary Video 3. Human pancreas vasculature.** 3-D extended projection of human pancreas exocrine and endocrine vasculature are shown with X, Y and Z axes in mm (Scale bar 200 µm). The extensive nature of the human pancreas vascular system is demonstrated by immunolabeling with monoclonal anti-CD31 (red) and islets are shown stained with monoclonal anti-glucagon (green) antibodies. The islets and microvasculature are shown individually as well as merged. The video shows a confocal image stack imaged using a 5x/0.16 objective on a Zeiss Z1 Lightsheet (342 slices imaged at 2.5 µm each, total 845 µm) and processed using Arivis software. Scale bar major ticks 200 µm.

**Supplementary Video 4. 3-D projection human primary pancreas exocrine cells in culture**. Isolated human exocrine cells were obtained from a pancreas donor non-islet fractions and following filtration to remove islets and clumps, exocrine cells were plated in 1:1 DMEM:F12 and Matrigel and grown for 6 days. A maximum projection image shows ductal cells (cytokeratin 19, green), acinar cells (amylase, red) and nuclei (blue). The video shows a confocal image stack imaged using a 40x/0.95 objective on a Zeiss LSM 710 (12 slices imaged at 0.97 µm each, total 12 µm). Cells were kindly provided by Dr. Thomas Schmittgen, College of Pharmacy, University of Florida. Scale bar 20 µm.

**Supplementary Video 5. Panoramic and high-resolution movie of optically cleared NOD mouse pancreas with insulitis.** Islets with insulitis from an 8-week NOD mouse are shown with blood vessels (red), lymphatic vessels (magenta), and T lymphocytes (green). The video shows a confocal image stack imaged using a 25x/0.8 objective on a Zeiss LSM 800 (41 slices imaged at 2.5 µm each, total 100 µm). Scale bar 200 µm.
